# Human T-Lymphotropic Virus-1 Visualized at the Virological Synapse by Electron Tomography

**DOI:** 10.1371/journal.pone.0002251

**Published:** 2008-05-28

**Authors:** Endre Majorovits, Mohamed Nejmeddine, Yuetsu Tanaka, Graham P. Taylor, Stephen D. Fuller, Charles R. M. Bangham

**Affiliations:** 1 Division of Structural Biology, Wellcome Trust Centre for Human Genetics, University of Oxford, Oxford, United Kingdom; 2 Department of Immunology, Wright-Fleming Institute, Imperial College London, London, United Kingdom; 3 Department of Immunology, Graduate School and Faculty of Medicine, University of the Ryukyus, Okinawa, Japan; 4 Department of Genito-Urinary (GU) Medicine and Communicable Diseases, Wright-Fleming Institute, Imperial College London, London, United Kingdom; Institut Pasteur, France

## Abstract

Human T-lymphotropic virus 1 (HTLV-1) is transmitted directly between cells via an organized cell-cell contact called a virological synapse (VS) [1], [2]. The VS has been studied by light microscopy, but the ultrastructure of the VS and the nature of the transmitted viral particle have remained unknown. Cell-free enveloped virions of HTLV-1 are undetectable in the serum of individuals infected with the human T-lymphotropic virus 1 (HTLV-1) and during in vitro culture of naturally infected lymphocytes. However, the viral envelope protein is required for infectivity of HTLV-1, suggesting that complete, enveloped HTLV-1 virions are transferred across the synapse. Here, we use electron tomography combined with immunostaining of viral protein to demonstrate the presence of enveloped HTLV-1 particles within the VS formed between naturally infected lymphocytes. We show in 3D that HTLV-1 particles can be detected in multiple synaptic clefts at different locations simultaneously within the same VS. The synaptic clefts are surrounded by the tightly apposed plasma membranes of the two cells. HTLV-1 virions can contact the recipient cell membrane before detaching from the infected cell. The results show that the HTLV-1 virological synapse that forms spontaneously between lymphocytes of HTLV-1 infected individuals allows direct cell-cell transmission of the virus by triggered, directional release of enveloped HTLV-1 particles into confined intercellular spaces.

## Introduction

Human T cell Lymphotropic Virus 1 (HTLV-1) is a complex retrovirus that causes Adult T cell Leukaemia/Lymphoma (ATL) [3] in 2–3% and several chronic inflammatory diseases in another 2–3% of infected people [4]–[7]. The main host cells infected by HTLV-1 in vivo are CD4^+^ and CD8^+^ T cells [5], [8]–[10]. Cell-cell contact is required for efficient transmission of HTLV-1, both within and between individuals [11], [12] and the virus is transmitted through an organized cell-cell contact termed the virological synapse (VS) [2]. The VS has a peripheral adhesion domain and one or several central transmission domains, similar to the adhesive peripheral supramolecular activation cluster (pSMAC) and the signalling central supramolecular activation cluster (cSMAC) in the immunological synapse (IS) [13]. Upon cell-cell contact between an HTLV-1-infected cell and a target cell, the virus induces a prolonged and strong adhesion. Expression of the viral transcriptional transactivator protein Tax and engagement of the cell adhesion molecule ICAM-1 cause the polarization of the microtubule organizing centre (MTOC) of the infected cell towards the cell-cell contact [14], [15]. The transport of viral material towards the VS and into the recipient cell requires the integrity of the microtubule cytoskeleton [2]. The HTLV-1 surface glycoprotein Env is required for infectivity [12], [16], suggesting that enveloped virions are transmitted between cells across the VS. However, HTLV-1 is typically undetectable in the serum of infected individuals by RT-PCR, and the apparent lack of extracellular virions has precluded detailed study of transmission from naturally infected cells. To identify the mode of viral transmission in the HTLV-1 VS we examined the ultrastructure of the VS using electron tomography of immunostained conjugates formed between CD4^+^ T cells from HTLV-1-infected individuals and conjugates between MS9 cells (a chronically HTLV-1-infected cell line) and Jurkat cells. The results reveal the ultrastructure of the HTLV-1 virological synapse and reconcile the requirement for cell contact and HTLV-1-Env protein for HTLV-1 infectivity with the lack of detection of cell-free virions in the serum.

## Results

### Two dimensional ultrastructure of HTLV-1 Virological synapse

To identify the mode of viral transmission in the HTLV-1 VS we examined the ultrastructure of the VS using electron tomography of cell-cell conjugates spontaneously formed either between autologous CD4^+^ T cells isolated from the PBMCs of HTLV-1-infected HAM/TSP individuals with high proviral load or between MS9 cells (a chronically HTLV-1-infected cell line), as a donor T-cell, and the Jurkat cell line, as a target T-cell. The use of electron tomography permits the reconstruction of a 3D image [17] from transmission electron micrographs of successive sections through the depth of a sample preparation. The cell-cell conjugate preparation was carried out under strict biosafety conditions. To preserve the cell integrity and the immediate environment of the cell-cell conjugates we used a chemical fixation protocol in which the cells were subjected to two steps of treatment: a short and gentle fixation “*in situ*” with a buffer solution containing paraformaldehyde (PFA) and glutaraldehyde, immediately followed by a second fixation for a longer time (see Materials and Methods section). Finally, the samples were post-fixed with osmium tetroxide and embedded in resin. In order to detect HTLV-1 virions at the VS, certain samples were stained with an antibody against the HTLV-1 Gag p19 matrix protein before embedding. Tilt series of 200–300 nm thick sections were recorded with an FEI Tecnai F30 transmission-electron microscope (300 kV) on a 2k×2k Gatan CCD camera at low magnification (∼10k) to capture the whole synapse and tomographic reconstructions were calculated using the software IMOD [18]. The tomographic data have been deposited in the Macromolecular Structure Database of the European Bioinformatics Institute (unique accession code EMD-1426).

**Figure 1:**
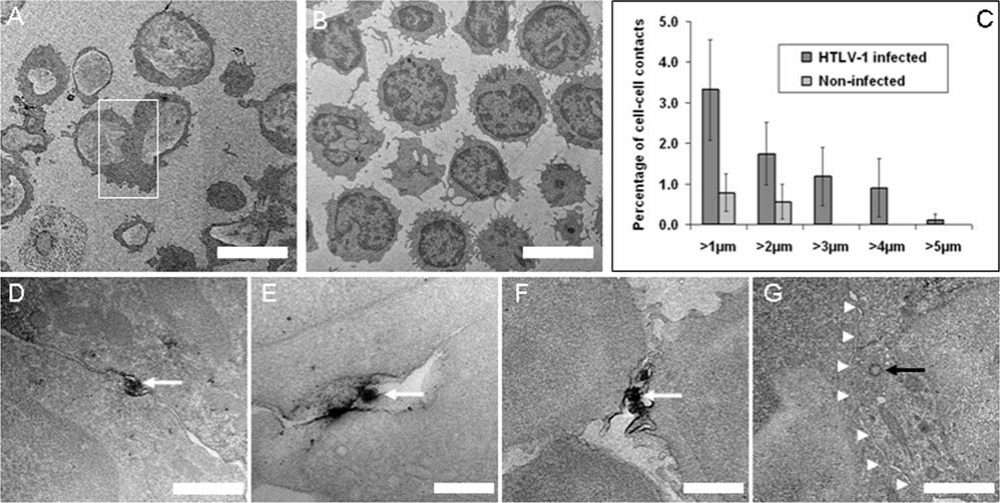
Two-dimensional ultrastructure of the HTLV-1 virological synapse. A, B Low-magnification images of single CD4+ T cells and cell-cell conjugates: A, naturally HTLV-1 infected CD4+ T cells (PBMCs); B, non-infected CD4+ T cells. Cell-cell conjugates were significantly more frequent and larger among HTLV-1 infected cells. C, frequency of cell-cell conjugates (relative to the total cell population) with a given minimum interface length. Membrane-membrane interfaces were measured on 2D images for HTLV-1 infected (black bars) and uninfected (grey bars) CD4+ T cells. Error bars: 90% confidence interval. D–G, Electron micrographs showing virological synapses between naturally HTLV-1 infected CD4+ T cells and target cells with different morphologies. Cells were labelled against the viral Gag p19 matrix protein and stained with DAB/HRP and OsO_4_. D–F, Darkly stained HTLV-1 particles, using the anti-Gag p19 monoclonal antibody, are held in the gap between the conjugated cells (white arrows). The enveloped particles are often touching one or both cell membranes. The virus transmission domain is usually surrounded by a peripheral adhesion domain which is characterized by close membrane apposition (D,E). G, Continuous cell-cell contact with polarized microtubule organizing centre (MTOC) as indicated by a centriole (black arrow) at the synapse (white arrow heads). Detail of image A. Scale bars: A, B 5000 nm, D 500 nm, E 200 nm, F, G 1000 nm.

We analysed a large number of randomly recorded low-magnification EM data sets of HTLV-1-infected (Figure 1A) and uninfected (Figure 1B) CD4^+^ T cells. The formation of cell-cell conjugates was significantly more frequent among HTLV-1 infected CD4^+^ T cells (2.8% conjugates in a total of 1084 cells) than in samples with non-infected CD4^+^ T cells (0.8% conjugates in 619 cells) (χ^2^ = 6.58 with Yates' correction; two-tailed P = 0.02). MS9 (HTLV-1-producing) cells were distinguished from uninfected (Jurkat) cells by the smaller diameter, the more uniform morphology and the better preserved intracellular structures in Jurkat cells. In conjugates formed among fresh PBMCs, infected cells were identified by positive staining for HTLV-1 Gag protein.

**Figure 2:**
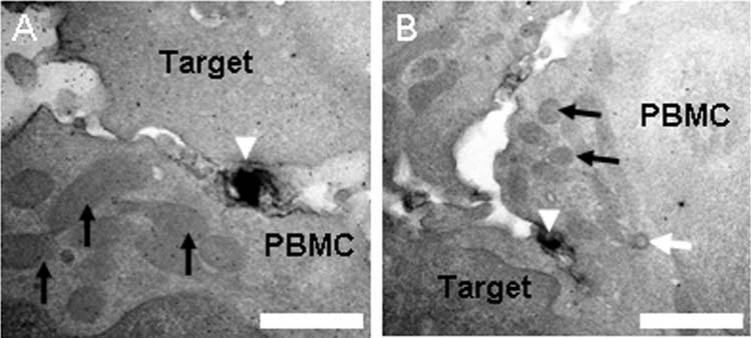
Polarization of mitochondria and microtubule organization centre of the HTLV-1 infected cell towards the target cell. A,B Virological synapses (VS) formed between naturally HTLV-1 infected CD4^+^ T cells (PBMCs) and autologous uninfected CD4^+^ target cells as shown in Movies S6–S7 and S3, S4, S5, respectively. HTLV-1 particles in the synaptic cleft can be identified by the black anti-Gag p19 labelling (white arrow heads). Mitochondria (black arrows) are located in the vicinity of the synapse; most mitochondria (black arrows) are polarized towards the VS. Also the MTOC is polarized to the synapse as revealed by one of the two centrioles (white arrow in B). Scale bars: A and B 500 nm.

The 2D measured length of the cell-cell contacts was greater in conjugates between HTLV-1 infected CD4^+^ T cells (mean, 2.8 µm; max., 5.6 µm) than in normal CD4^+^ T cell conjugates (mean, 1.9 µm; max., 2.5 µm) (Figure 1C). In a sample of 1084 CD4^+^ T cells stained for HTLV-1 Gag protein we found 7 (0.65%) cell-cell conjugates with anti-Gag labelling at the VS (Figure 1D,E,F, arrows). HTLV-1 particles were identified by their close resemblance in size and morphology to the HTLV-1 particles produced by in vitro cell lines, described by Miyauchi et al [19], and by positive staining for Gag protein; such particles were not observed in uninfected cells or conjugates. Many HTLV-1 particles were almost entirely surrounded by the adjacent cell membranes (Figure 1D); others were contained in a larger synaptic pocket (Figure 1E). In several cases, viral particles were in contact with the two adjacent cell membranes simultaneously (Figure 1D,F). Mitochondria frequently accumulated in the infected cell in the vicinity of the VS (Movies S1, Movie S3, Movie S6; Figure 2A,B), consistent with the polarization of the MTOC to the VS [2], [14], [15] (Figure 1G, arrow pointing at centriole), as seen at the immunological synapse [20]–[22].

### Intermembrane spacing in the virological synapse and the size of HTLV-1 virions

**Figure 3:**
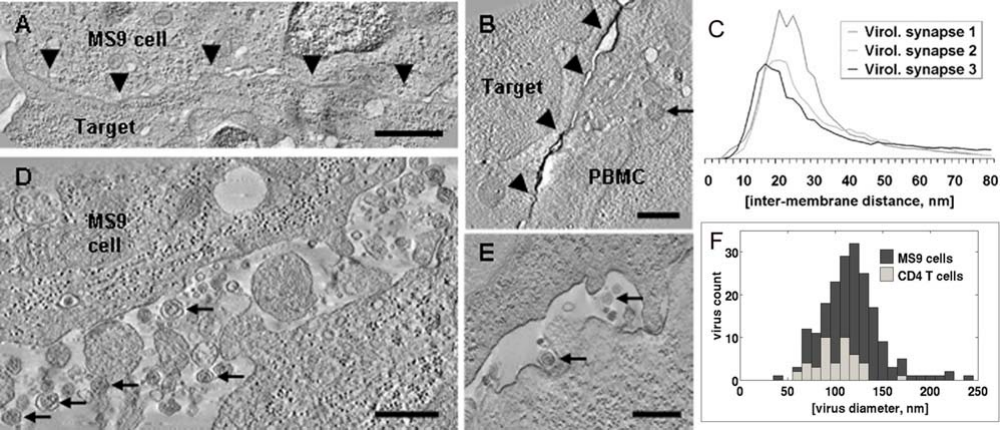
VS membrane-membrane distances and HTLV-1 virion diameter. A, B Tomogram slices showing close membrane apposition in the HTLV-1 virological synapse (VS) (black arrowheads): A, VS formed between an HTLV-1 infected MS9 cell as a donor and Jurkat cell as target. B, VS formed between a CD4^+^ T-cell naturally infected with HTLV-1 (PBMC) and an autologous uninfected CD4^+^ as a target cell. These cells were stained against HTLV-1 Gag p19 matrix protein with a specific monoclonal antibody (GIN7). The microtubule organizing centre is polarized to the synapse as revealed by one of the two centrioles (black arrow). C, The spacings between those areas of the conjugated cell membranes that constitute the VS were measured in three VS between naturally HTLV-1 infected CD4^+^ T-cell (PBMC) and autologous uninfected CD4^+^ as target cells: each distribution shows a peak around 20 nm. D, Tomogram slice showing a large number of virus particles (examples indicated by black arrows) that budded from an MS9 cell into the extra-cellular space. E, Tomogram slice showing virions (two examples indicated by black arrows) that budded from a naturally HTLV-1 infected CD4^+^ T-cell (PBMC) into the synaptic cleft. Most of these virions are not attached to a cell membrane. F, Distribution of diameter of HTLV-1 virions from MS9 cells (dark grey) and naturally infected CD4^+^ T cells (light grey): the distributions are slightly different with a median of 124 nm and 108 nm, respectively. Scale bars: A 1000 nm, B,D,E 500 nm.

We used the tomographic data to measure the intermembrane spacing in the VS and the diameter of HTLV-1 particles. Areas of continuous and close apposition of the plasma membranes were present at the cell-cell contact (Figure 3A,B). The membrane spacing in the adhesion area in three synapses showed a distribution with a strong peak at ∼20 nm and an averaged median value of 25.7 nm (Figure 3C). HTLV-1 particles derived from naturally infected CD4^+^ T cells (N = 44) had a mean diameter of 105 nm (range 62–173 nm) and a median of 108 nm (Figure 3E,F). Particles derived from the MS9 cell line (N = 151) had a mean diameter of 126 nm (range 46–246 nm) and a median of 124 nm (Figure 3D,F).

### The three dimensional ultrastructure of the HTLV-1 virological synapse reveals the presence of virions in intercellular synaptic clefts

**Figure 4:**
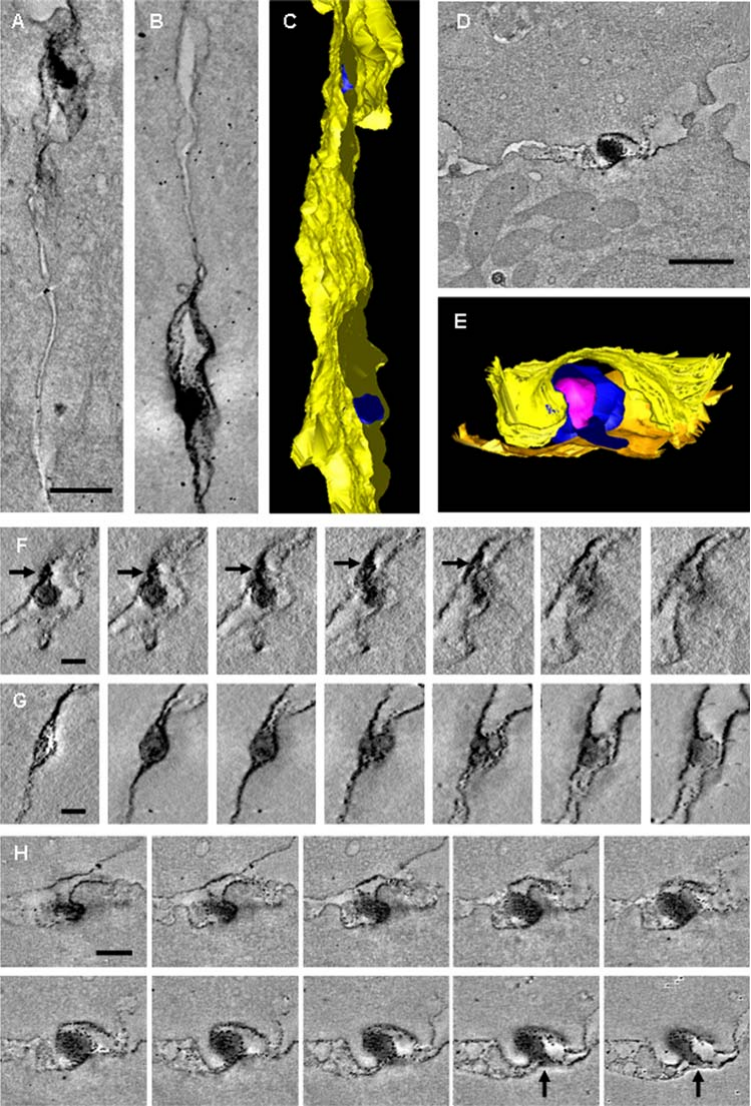
Three-dimensional ultrastructure of the VS: enveloped HTLV-1 virions are trapped in multiple isolated synaptic clefts. Cell-to-cell transmission of HTLV-1 as observed in tomograms of the VS formed between HTLV-1 infected CD4^+^ T-cell (PBMC) and an autologous uninfected CD4^+^ T-cell as a target cell. These cells were stained against HTLV-1 Gag p19 matrix protein with a specific monoclonal antibody (GIN7). A, B, Projections along the z-axis of two subvolumes of the same tomogram showing viral transmission at two different locations. C, Surface representation of the VS shown in (A, B): Several virions (blue) are trapped between the closely apposed plasma membranes (yellow). D, Tomogram slice showing an HTLV-1 particle held between the cell membranes. E, Surface representation of the virus transmission shown in D (cell membranes: yellow and orange, virus envelope: blue, virus core: magenta). F, G, Tomogram slices through the two areas of virus transmission shown in (A) and (B), respectively, with a spacing of about 17 nm (F) and 25 nm (G) between subsequent slices. Black arrows indicate a protrusion linking the virus with the cell membrane. H, Subsequent slices through the area of virus transmission shown in (D) with a spacing of about 17 nm. Black arrows indicate a protrusion linking the virus with the cell membrane. Scale bars: A,B 300 nm, D 500 nm, F,G 100 nm, H 200 nm.

**Figure 5:**
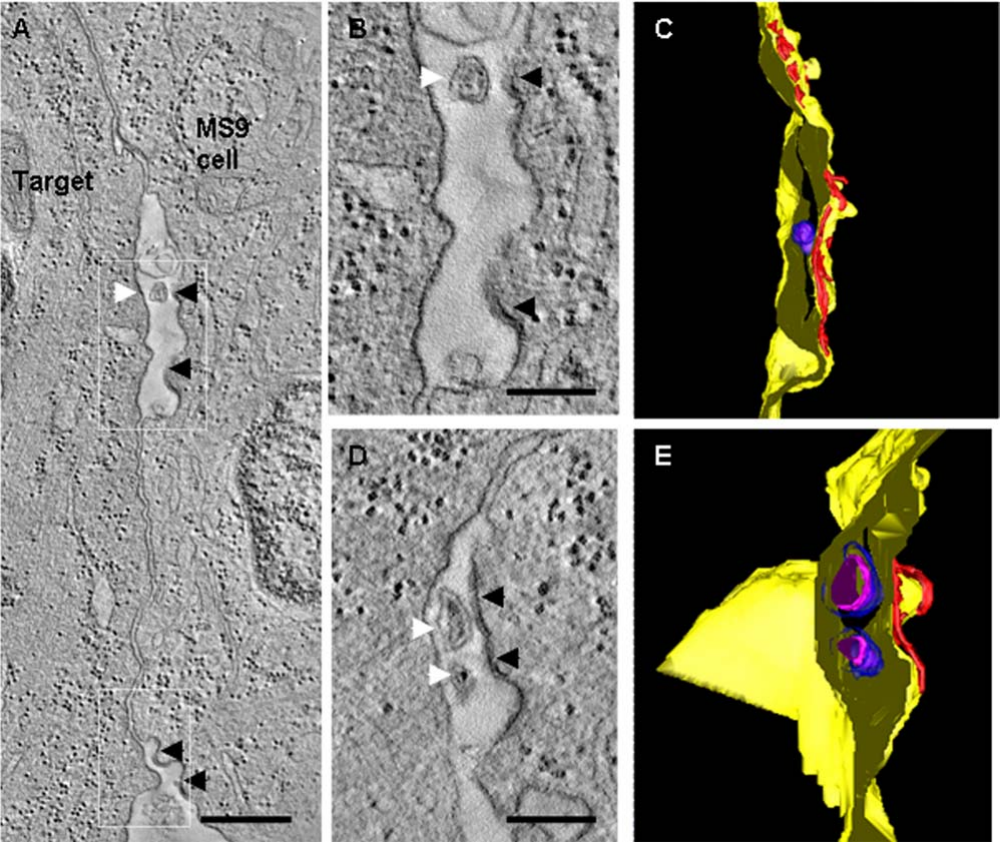
Tomogram slices and surface representations of the virological synapse between an HTLV-1 infected MS9 cell and a target cell. A, The VS is characterized by a tight membrane-membrane contact with an inter-membrane spacing of about 20 nm. Viral budding sites (black arrowheads) and virus particles (white arrowhead) can be detected within a synaptic pocket and at the periphery of the synapse formed between HTLV-1 infected MS9 cell as a donor, and Jurkat cell as a target. B and D show slices through the synaptic cleft and the periphery, respectively, as indicated by the white rectangles in (A), C and E show the corresponding surface representations (cell membranes: yellow, virus envelope: blue, virus core: magenta, viral core protein at budding site: red). Scale bars: A 500 nm, B and C 200 nm.

Conjugates formed with naturally infected CD4^+^ T cells contained small numbers of isolated, cell-free virus particles (Figure 3E and Movie S2). However, most HTLV-1 particles were in contact with a cell membrane. We observed Gag staining associated with both cell membranes in the synaptic cleft, consistent with the transfer of viral protein from cell to cell (Figure 3B). Successive tomogram slices (Figure 4F,G,H) showed that HTLV-1 particles frequently touched both cell membranes at the same time (see also Figure 4E and Movies S3, Movie S4, Movie S5, Movie S6, Movie S7). These results suggest that the virion can fuse with the recipient cell membrane before complete detachment from the infected cell. We also found that virus budding into a synaptic pocket was not limited to one location, but occurred at different places (i.e. spatially separated synaptic pockets) simultaneously in the same synapse (Figure 4A,B,C, Movie S3). In contrast with these observations on naturally-infected lymphocytes, VS formed between target cells and the continuous HTLV-1-producer cell line MS9 [14] showed sites of virus budding and abundant virus particles both inside the VS (Figure 5A,B,C and Movies S8, S9), and more prominently at the periphery, i.e. outside the synapse (Figure 3D, Figure 5A,D,E and Movie S1).

## Discussion

**Figure 6:**
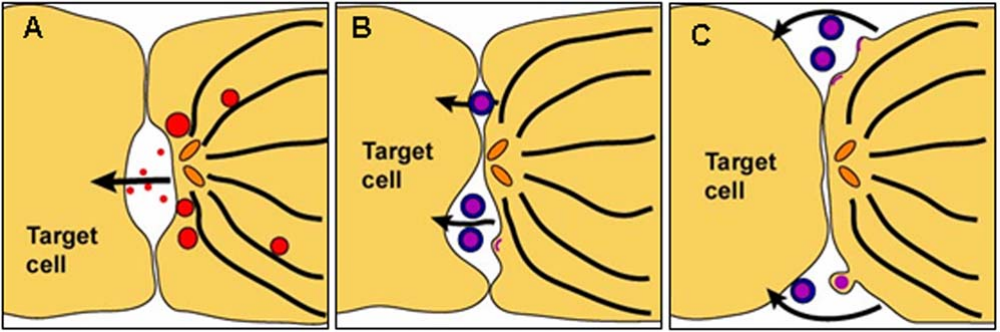
Models of immunological and virological synapses. A, Upon formation of an IS between a cytotoxic T lymphocyte (CTL) and a target cell the microtubule organizing centre is polarized towards the synapse (the centrioles are depicted as orange ellipses). Lytic granules containing cytotoxins (red discs within CTL) are transported along microtubules (black lines) to the synaptic cleft. The cytotoxins (red points) are released into the synaptic cleft and taken up by the target cell. B–C, Possible modes of viral transmission through the HTLV-1 virological synapse: B, Enveloped HTLV-1 particles (purple-blue discs) bud into the synaptic cleft and are transmitted to the target cell. C, Enveloped virus particles bud at the periphery of the synapse and are transmitted through the extracellular space.

Exogenous retroviruses contain an envelope, derived from the plasma membrane of the infected cell, which undergoes fusion with the plasma membrane of the target cell mediated by the viral surface glycoprotein Env. Like other retroviruses, HTLV-1 requires Env for infectivity, suggesting that HTLV-1 spreads between cells as conventional enveloped particles. However, HTLV-1 spreads directly between cells via an organized cell-cell contact called a virological synapse [2]. Furthermore, cell-free HTLV-1 particles are usually undetectable in the serum of HTLV-1-infected subjects, and cell-free blood products are not infectious. Our tomographic data reconcile these observations by showing that enveloped HTLV-1 particles are present in an intercellular synaptic cleft in the VS between naturally infected lymphocytes which is characteristically bounded by the tightly apposed membranes of the infected and target cells. In some cases, the virus particle detaches from the infected cell before making contact with the target cell (Figure 6B, bottom). More commonly, however, virus particles were observed in contact with the infected cell, with the target cell, or with both cells simultaneously (Figure 6B, top). The continuously infected producer cell line, MS9, also showed virions at the periphery of the synapse. These particles may remain visible under EM because they fail to fuse with the plasma membrane of the target cell. However, virtually no HTLV-1 particles from naturally infected CD4^+^ T cells were found at the periphery of the VS (Figure 6C).

It was previously shown that HTLV-1 induces cell-cell fusion in certain cell lines in vitro [23], raising the possibility that HTLV-1 material is transferred across cell-cell membrane bridges in vivo. However, we did not observe fusion between the two cell membranes themselves. Our results therefore favour the model shown in Figure 6B. These results suggest that there is rapid transfer of enveloped HTLV-1 particles from cell to cell across the VS. The directional nature of the process is indicated by the polarization of Gag protein [2], [14], [15] and the restriction of budding and released virions to the cell-cell contact area in the VS formed with naturally-infected cells.

Microtubule polarization in the IS [20] is triggered by engagement of the T-cell receptor, whereas in the VS the polarization is triggered by a synergistic interaction between the HTLV-1 Tax protein and ICAM-1 engaged on the infected cell surface [14], [15]. The differences between the VS and the IS are extended here: whereas secretory lysosomes are secreted into a single synaptic pocket in the cytotoxic synapse [21], [22] (Figure 6A), the tomographic data of the HTLV-1 VS (Figure 4C, Figure 6B) demonstrated multiple, spatially distinct sites of virus budding into synaptic pockets that are separated by areas of close membrane–membrane apposition. These adhesion areas are characterized by a membrane-membrane distance of ∼20 nm (averaged median value of 25.7 nm). This finding is consistent with previous measurements of cell-cell separation between lymphocytes and target cells [24], and with data on the conformation of ICAM-1/LFA-1 binding from electron microscopy [25] and X-ray crystallography [26]. The size of HTLV-1 particles that originate from naturally infected CD4^+^ T cells has a wide range (62 nm–173 nm), similar to the variability in size of HIV-1 particles [27], [28], with a peak at about 100 nm. The size distribution of particles derived from the MS9 cell line was similar but the mean size of MS9-derived particles was slightly larger. Our results are similar to previous measurements of HTLV-1 particles obtained from two different cell lines [19].

Whereas most viruses spread by releasing large numbers of virions from the infected cell, HTLV-1 uses the mobility of the host cell to propagate from cell to cell. Other synapse-inducing retroviruses such as HIV-1 [5], [29] may use a similar mechanism of viral transmission. Sequestration in the synaptic cleft presumably allows efficient transfer of small numbers of virions, and may give the virions a degree of protection from components of the immune response (complement system and antibodies). We conclude that the HTLV-1 VS allows efficient propagation of HTLV-1 by triggered, directional transfer of enveloped virions directly from cell to cell.

## Materials and Methods

### Naturally infected CD4^+^ T cells

Peripheral blood mononuclear cells (PBMCs) were donated by HTLV-1 seropositive patients with HTLV-1-associated myelopathy/tropical spastic paraparesis (HAM/TSP) attending the National Centre for Human Retrovirology at St Mary's Hospital, London.

We used samples from three different patients with HAM/TSP, each with a high proviral load of HTLV-1: one sample was not immunostained and the two others were stained with anti-Gag antibody. We also used CD4^+^ T-cells isolated from an HTLV-1 seronegative healthy donor as a negative control.

All patients gave written informed consent, and the study was approved by the St Mary's NHS Trust Local Research Ethics Committee. The PBMCs were isolated by density gradient centrifugation on Histopaque-1077 (Sigma-Aldrich Company Ltd, Dorset, UK), washed twice with phosphate buffered saline (PBS) and washed once in PBS/10% FCS. CD4^+^ T cells were negatively selected from the PBMCs using the CD4^+^ T cell isolation Kit from Miltenyi Biotech, Surrey, UK, following the manufacturer's instructions. This procedure yielded CD4^+^ cells at a purity of greater than 90%, ascertained by flow cytometry (data not shown). Before use, the isolated CD4^+^ T cells were cultured overnight in 10 cm diameter tissue culture dishes (10^6^ cells/ml), to allow spontaneous expression of HTLV-1 proteins [30]. During this incubation, the cells were widely dispersed to minimize cell-cell contact. Before processing for EM the cells were gently centrifuged for 5 minutes at 300 g to induce cell-cell conjugates and then incubated for another 1 h at 37°C and 5% CO_2_.

T cells were cultured in RPMI 1640 medium (Sigma-Aldrich Company Ltd, Dorset, UK) supplemented with 2 mM glutamine (Invitrogen Ltd, Paisley, UK), 100 IU/ml penicillin (Invitrogen Ltd, Paisley, UK), 100 IU/ml streptomycin (Invitrogen Ltd, Paisley, UK) and 10% heat-inactivated fetal calf serum (PAA Laboratories Ltd, Somerset, UK).

### HTLV-1-immortalized cell line and Jurkat cell line

The HTLV-1- immortalized cell line, MS9 was a gift from Dr. David Derse, National Cancer Institute, Maryland, USA. MS9 cells were derived by co-culture of phorbol-12- myristate-13-acetate-activated human peripheral PBMCs with DBS-FRhL (clone B5) cells that were infected with the HTLV-1 molecular clone, pHTLV-X1MT [31]. MS9 cells were cultured in RPMI 1640 (Sigma-Aldrich Company Ltd, Dorset, UK) supplemented with 2 mM glutamine (Invitrogen Ltd, Paisley, UK), 100 IU/ml penicillin (Invitrogen Ltd, Paisley, UK), 100 IU/ml streptomycin (Invitrogen Ltd, Paisley, UK), 20% heat-inactivated fetal calf serum (PAA Laboratories Ltd, Somerset, UK) and 100 U/ml recombinant interleukin 2 (IL-2; Sigma-Aldrich Company Ltd, Dorset, UK). Jurkat cells (clone E6.1) were obtained from ATCC, Middlesex, UK. Jurkat E6.1 is a clone of the Jurkat-FHCRC cell line, a derivative of the Jurkat cell line [32]. Before processing for EM and/or IFM the cells were mixed 1∶1 with Jurkat cells and incubated for 30 minutes at 37°C and 5% CO_2_ to induce cell-cell conjugates.

### Sample preparation for EM

Samples were prepared with different buffers and different amounts of fixatives, depending on whether they were destined to be immunostained with the mAb anti-Gag p19 (GIN7) or to be stained with magnesium uranyl acetate (Sigma-Aldrich Company Ltd, Dorset, UK). In general, the fixation of the samples was performed sequentially in two distinct fixative solutions: Solution A, 2% paraformaldehyde (PFA) (Electron Microscopy Science, Hatfield, PA, USA) and 0.5% glutaraldehyde (Agar Scientific Ltd, Essex, UK) in sodium cacodylate buffer 0.1 M, pH 7.2 (Sigma-Aldrich Ltd, Dorset, UK); and Solution B, 1% osmium tetroxide (OsO_4_) in PBS (Sigma-Aldrich Ltd, Dorset, UK).

After incubating the samples to induce cell-cell conjugates, they were pre-fixed in the 10× diluted PFA/glutaraldehyde (Solution A) for 10 minutes before centrifugation. After centrifuging for 5 minutes at 1000 g the samples were fixed for another hour with the undiluted fixative (Solution A). The samples were washed 3 times either with PBS 1% BSA or with sodium cacodylate buffer 0.1 M, pH 7.2, and then processed for antibody staining as described below or post-fixed with 1% OsO_4_ (Solution B) and stained in magnesium uranyl acetate overnight, respectively.

The fixed samples were dehydrated through a series of ethanol exchanges and embedded in Agar 100 resin (Agar Scientific Ltd., Stansted, UK). The samples were baked at 60°C overnight to give solid blocks that were sectioned with an ultramicrotome (Leica Ultracut UCT, Leica Microsystems GmbH, Wetzlar, Germany) at a thickness of 60–300 nm. Thin samples (60–100 nm) were floated onto 200/300 mesh square Ni grids (Agar Scientific Ltd., Stansted, UK). Thick samples for tomography (200–300 nm) were either floated onto 200 mesh square Ni grids or onto formvar film coated Cu 1×2 mm slot grids (Agar Scientific Ltd., Stansted, UK) to allow for the collection of serial sections. Samples that were not antibody labelled were stained with lead citrate (Leica Ultrostain2) for 3–10 minutes, depending on the thickness of the sections, by floating the grids on drops of the staining solution. For electron tomography the grids were covered with 10 nm or 15 nm fiducial gold beads (Sigma-Aldrich Company Ltd, Dorset, UK) to facilitate image processing.

### Pre-embedding antibody staining for EM

To detect HTLV-1 Gag matrix protein in infected cells the samples were labelled against HTLV-1 Gag p19 [33] using the DAB kit from Molecular Probes (Invitrogen Ltd, Paisley, UK ). After the PFA/glutaraldehyde fixation, the samples were incubated in PBS containing 1% BSA, 0.1% Triton X-100 and 2% H_2_O_2,_ for 1 h at 37°C to block non-specific antibody binding and to quench the endogenous peroxidase activity of the cells. To detect the HTLV-1 complexes, the cells were incubated with 5 µg/ml of anti-Gag p19 (GIN7 mAb) [33] diluted in PBS 1% BSA, for 1 h at 37°C. Then the second antibody, anti-Mouse mAb conjugated to HRP (Invitrogen Ltd, Paisley, UK ), was added at a final dilution of 1 µg/ml, and incubated for 30 minutes at 37°C. The cells were washed three times with PBS containing 1% BSA between each step.

The sample was centrifuged and embedded into a 1% agarose gel (Sigma-Aldrich Ltd, Dorset, UK). A 1 mg/ml DAB solution (Invitrogen Ltd, Paisley, UK ) diluted in PBS/1%BSA was added to the samples and incubated for 15 minutes at room temperature with gentle shaking. The staining was detected by adding to the samples 0.03% H_2_O_2_ diluted in PBS/1%BSA. The samples turned brown within 30 s to 5 min and the reaction was then stopped by washing extensively with PBS. Between each procedure the cells were centrifuged at 1000 g and washed thoroughly at least three times in PBS. The samples were finally fixed for at least 5 minutes in a 1% OsO_4_ solution (samples turned black) and washed 5 times before further processing.

### Electron microscopy, tomography, modelling and data analysis

Electron microscopic data were collected with an FEI Tecnai F30 FEG transmission electron microscope (FEG-TEM) (FEI Company, Eindhoven, The Netherlands) at an electron voltage of 300 kV. The TEM was equipped with an energy filter (Gatan imaging filter (GIF), Gatan, Inc., Abingdon, UK). Electron micrographs were recorded using the software Digital Micrograph (Gatan, Inc., Abingdon, UK) with a Gatan 2k×2k slow-scan CCD camera. Most tomographic data sets were recorded at a few microns under focus using the GIF with a slit width of 20–25 eV to accentuate contrast. Tilt series for tomography were recorded in steps of 1° over a tilt range of typically −65° to +65° using the FEI software. Most tomographic data sets were recorded using a flip-flop sample holder (Gatan) that allows one to turn the sample by 90° inside the EM column. A few data sets were recorded in the Laboratory for 3-Dimensional Electron Microscopy of Cells in Boulder, Colorado with a FEI Tecnai F30 FEG-TEM (without energy filter). These data were recorded using the software SerialEM (Boulder, Colorado) with a Gatan CCD camera. Tomograms were processed with the tomography software IMOD [34] and surface representations were generated using both IMOD and the software package AMIRA (Mercury Computer Systems, Inc.). Data analysis was performed using IMOD, AMIRA and Matlab (The MathWorks). Membrane-membrane spacings were measured using a program that calculates the distance between each voxel of the digitized membrane model (surface representation) and its nearest neighbour in the opposite cell membrane. The size of individual HTLV-1 particles was determined by measuring an averaged diameter in the section (through the 3D object) of largest appearance of the particle.

## Supporting Information

Movie S1 Tomographic reconstruction showing a large number of HTLV-1 particles of variable size in the space between a chronically infected MS9 cell (top) and a potential Jurkat target cell (bottom). The particles are characterized by an electron-dense core surrounded by a less dense area and enveloped by the electron-dense viral membrane. The average diameter of HTLV-1 particles derived from MS9 cells was 126.0 +/− 31.3 nm (SD). White arrows indicate areas in the MS9 cell where viral capsid protein accumulates below the membrane in anticipation of virus budding. Three small parts of mitochondria (upper left corner) and several microtubules are visible in the MS9 cell cytoplasm. A slice through the entire tomogram is shown in Figure 3D. The sample was fixed with PFA/glutaraldehyde, embedded in Agar 100 resin, post-fixed with OsO4 and stained with lead citrate. The reconstructed 3D volume is composed of tomograms obtained from four serial sections. The tomograms were calculated from tilt series of 121 images (tilt range −60° to +60°, 1° increment). The images were recorded with a 2k×2k Gatan CCD camera (pixel size 1.21 nm) at an under focus of −0.2 µm with an FEI Tecnai F30 FEG-TEM (300 kV) in Boulder, Colorado using the software SerialEM. The reconstruction was calculated using the software IMOD. The tomogram represents a volume with an area of 1.94×1.28 µm2 and a thickness of 640 nm. The scale bar at the end of the movie corresponds to 400 nm.
(1.35 MB MPG)

Movie S2 Tomographic reconstruction showing a few virus particles that budded from a naturally HTLV-1 infected CD4+ T cell (PBMC) into the synaptic cleft. Most of the virions are not attached to a cell membrane. The particles are characterized by an electron-dense core surrounded by a less dense area and enveloped by the electron-dense viral membrane. The average diameter of HTLV-1 particles derived from naturally HTLV-1 infected CD4+ T-cell (PBMC) was 105.2 +/− 21.8 nm (SD). A slice through the entire tomogram is shown in Figure 3E. The sample was fixed with PFA/glutaraldehyde, embedded in Agar 100 resin, post-fixed with OsO4 and stained with lead citrate. The tomogram was calculated from a tilt series of 141 images (tilt range −70° to +70°, 1° increment). The images were recorded with a 2k×2k Gatan CCD camera (pixel size 1.21 nm) at an under focus of −0.2 µm with an FEI Tecnai F30 FEG-TEM (300 kV) in Boulder, Colorado using the software SerialEM. The reconstruction was calculated using the software IMOD. The tomogram represents a volume with an area of 1.70×2.24 µm2 and a thickness of 176 nm.
(8.45 MB MPG)

Movie S3 Tomographic reconstruction showing the virological synapse between a naturally HTLV-1-infected CD4+ T-cell (PBMC) (right) and a target cell (autologous uninfected CD4+ T-cell) (left). Virus transmission can be seen in two separate synaptic clefts. The HTLV-1 particles are in simultaneous contact with the two cell membranes and are extremely electron-dense due to the labelling of the viral Gag p19 matrix protein (see also Movies S4 and S5). Parts of the two plasma membranes are also darkly stained, indicating the presence of viral Gag protein. The presence of two centrioles (long white arrows), two Golgi apparatuses (short, thick white arrows) and several mitochondria close to the VS reflects the polarization of the microtubule organizing centre (MTOC) of the HTLV-1-infected CD4+ T cell towards the cell-cell contact. Parts of the cell nucleus can be seen in both cells. Tomogram slices through the 3D reconstruction can be seen in Figure 4A,B and a surface representation of the synapse is shown in Figure 4C. The sample was fixed with PFA/glutaraldehyde, labelled against viral Gag p19 matrix protein, embedded in Agar 100 resin and post-fixed with OsO4. The reconstructed 3D volume is composed of tomograms obtained from five serial sections. The tomograms were calculated from tilt series of 131 images (tilt range −65° to +65°, 1° increment). The images were recorded with a 2k×2k Gatan CCD camera (pixel size 1.29 nm) at an under focus of −3.0 µm with an FEI Tecnai F30 FEG-TEM (300 kV) using the FEI software. The reconstruction was calculated using the software IMOD. The tomogram represents a volume with an area of 1.55×2.66 µm2 and a thickness of 343 nm. The scale bar at the end of the movie corresponds to 500 nm.
(6.59 MB MPG)

Movie S4 Detail of the tomographic reconstruction shown in Movie S3 illustrating an HTLV-1 particle trapped between the cell membranes. The enveloped virion touches both cell membranes. Some dense material links the particle with the target membrane (black area above the particle). See also Figure 4D. For technical details see legend of Movie S3.
(9.17 MB MPG)

Movie S5 Detail of the tomographic reconstruction shown in movie 3A illustrating HTLV-1 particles trapped between the cell membranes. The enveloped virions touch both cell membranes. See also Figure 4G. For technical details see legend of Movie S3.
(6.84 MB MPG)

Movie S6 Tomographic reconstruction showing the virological synapse between a naturally HTLV-1-infected CD4+ T-cell (bottom) and an autologous uninfected CD4+ T-cell cell, target cell (top). A large HTLV-1 particle (black arrow) is held between the two cell membranes and is extremely electron-dense due to the labelling against viral Gag p19 matrix protein (see also Movie S7). Parts of the two plasma membranes and inter-membrane material are also darkly stained, indicating the presence of abundant viral Gag protein. Several mitochondria have accumulated at the VS within the HTLV-1-infected CD4+ T (PBMC) which is characteristic of MTOC polarization, and is also seen in the IS. A tomogram slice showing a larger area of the 3D reconstruction can be seen in Figure 4D. The sample was fixed with PFA/glutaraldehyde, labelled against viral Gag p19 matrix protein, embedded in Agar 100 resin and post-fixed with OsO4. The reconstructed 3D volume is composed of tomograms obtained from five serial sections. The tomograms were calculated from tilt series of typically 131 images (tilt range −65° to +65°, 1° increment). The images were recorded with a 2k×2k Gatan CCD camera (pixel size 1.29 nm) at an under focus of −5.0 µm with an FEI Tecnai F30 FEG-TEM (300 kV) using the FEI software. The reconstruction was calculated using the software IMOD. The tomogram represents a volume with an area of 2.24×0.98 µm2 and a thickness of 313 nm. The scale bar at the end of the movie corresponds to 500 nm.
(4.18 MB MPG)

Movie S7 Detail of the tomographic reconstruction shown in Movie S6 illustrating an HTLV-1 particle held between the cell membranes. The enveloped virion touches both cell membranes simultaneously. Some dense material links the particle with the target membrane (black area in the bottom right of the particle at the end of the movie). See also Figure 4H. For technical details see legend of Movie S6.
(6.83 MB MPG)

Movie S8 Tomographic reconstruction showing a synaptic cleft formed between a chronically HTLV-1-infected MS9 cell (right) and a target Jurkat cell (left). The synaptic cleft is completely enclosed by a close membrane-membrane contact extending over a large area with a diameter of about 6 µm (see Figure 3A and Figure 5A). The membrane-membrane distance in areas of close apposition is about 20 nm. Two HTLV-1 particles are in the synaptic cleft (white arrowheads). White arrows indicate areas in the MS9 cell where viral capsid protein has accumulated below the membrane before directed virus budding. A tomogram slice showing a larger area of the 3D reconstruction can be seen in figure 5A. The sample was fixed with PFA/glutaraldehyde, embedded in Agar 100 resin, post-fixed with OsO4 and stained with lead citrate. The reconstructed 3D volume is composed of tomograms obtained from two serial sections. The tomograms were calculated from tilt series of typically 121 images (tilt range −60° to +60°, 1° degree increment). The images were recorded with a 2k×2k Gatan CCD camera (pixel size 1.54 nm) at an under focus of −0.2 µm with an FEI Tecnai F30 FEG-TEM (300 kV) in Boulder, Colorado using the software SerialEM. The reconstruction was calculated using the software IMOD. The tomogram represents a volume with an area of 1.32×1.92 µm2 and a thickness of 326 nm. The scale bar at the end of the movie corresponds to 500 nm.
(2.09 MB MPG)

Movie S9 Surface representation of the synaptic cleft between a chronically HTLV-1-infected MS9 cell and a Jurkat target cell shown in tomogram 5a. The cell membranes are depicted in yellow and HTLV-1 particles in blue. The red areas represent membrane segments within the MS9 cell where viral capsid protein has accumulated in anticipation of directed virus budding. The surface representation was generated using the software packages IMOD and AMIRA.
(9.80 MB MPG)
